# Src and SHP2 coordinately regulate the dynamics and organization of vimentin filaments during cell migration

**DOI:** 10.1038/s41388-019-0705-x

**Published:** 2019-01-29

**Authors:** Cheng-Yi Yang, Po-Wei Chang, Wen-Hsin Hsu, Hsuan-Chia Chang, Chien-Lin Chen, Chien-Chen Lai, Wen-Tai Chiu, Hong-Chen Chen

**Affiliations:** 1 Department of Life Sciences, National Chung Hsing University, Taichung, Taiwan; 2 Cancer Progression Research Center, National Yang-Ming University, Taipei, Taiwan; 3 Institute of Biochemistry and Molecular Biology, National Yang-Ming University, Taipei, Taiwan; 4 Institute of Molecular Biology, National Chung Hsing University, Taichung, Taiwan; 5 Department of Biomedical Engineering, National Cheng Kung University, Tainan, Taiwan

**Keywords:** Intermediate filaments, Phosphorylation

## Abstract

Vimentin intermediate filaments (VIFs), expressed in most mesenchymal and cancer cells, undergo dramatic reorganization during cell migration; however, the mechanism remains obscure. This study demonstrates that upon growth-factor stimulation, Src directly phosphorylates vimentin at Tyr117, leading to VIF disassembly into squiggles and particles at the cell edge during lamellipodia formation. The protein tyrosine phosphatase SHP2 counteracted the Src effects on VIF tyrosine phosphorylation and organization. VIFs formed by vimentin Y117D mutant were more soluble and dynamic than those formed by the wild-type and Y117F mutant. Increased expression of vimentin promoted growth-factor induced lamellipodia formation and cell migration, whereas the mutants suppressed both. The vimentin-induced increase in lamellipodia formation correlated with the activation of Rac and Vav2, with the latter associated with VIFs and recruited to the plasma membrane upon growth-factor stimulation. These results reveal a novel mechanism for regulating VIF dynamics through Src and SHP2 and demonstrate that proper VIF dynamics are important for Rac activation and cell migration.

## Introduction

The cytoskeleton is constituted by intermediate filaments (IFs), actin filaments, and microtubules, which are in intimate communication with one another [1]. Unlike actin filaments and microtubules, which exhibit the structural polarity, IFs are nonpolar structures that provide mechano-protection and regulate a broad range of cellular functions [2–5]. Whereas actin filaments and microtubules have few subtypes (α, β, and γ), ~70 genes in the human genome encode IF proteins [6]. IF proteins share a similar structural organization, which contains a central alpha-helical rod domain franked by a non-helical NH2-terminal head domain and a non-helical COOH-terminal tail domain. IFs can be classified into five major types according to their gene sub-structure and sequence homology within the central rod domain. The expression of IFs is tissue type- and differentiation program-dependent [7–10].

Vimentin, a 57-kDa protein, belongs to the type III IF proteins and is expressed in mesenchymal cell types, such as fibroblasts and bone marrow-derived blood cell lineages [11, 12]. Vimentin is strongly up-regulated following injury to various tissues and during the epithelial-mesenchymal transition [13, 14]. In addition to maintaining the structure of the cell, vimentin intermediate filaments (VIFs) exert pleiotropic and context-dependent functions and particularly have marked impacts on cell adhesion, motility, and invasion [15–19]. During cell migration, sheet-like membrane protrusion called lamellipodia is often formed at the leading edge of the cell. Increasing evidence indicates that VIFs are important for lamellipodia formation and cell-polarity maintenance in migrating cells. In a moving fibroblast, VIFs are disassembled into particles and short filament pieces called squiggles at the lamellipodia [20]. This disassembly at the lamellipodia is not observed in serum-starved cells or in non-migratory fibroblasts. Instead, serum-starved cells form a well network of VIFs that extends to the cell periphery. Furthermore, it has been reported that the disassembly of VIFs at the lamellipodia can be regulated by Rac1 [20], a small G protein in the Rho family that drives actin polymerization and mediates lamellipodia formation [21]. In addition, p21-activated kinase (PAK), a downstream effector protein for Rac and Cdc42 [22], has been shown to phosphorylate vimentin and induce VIF reorganization [23, 24].

The spontaneous self-assembly of VIFs can be triggered in vitro by increasing the salt concentration [2, 25]. At the initial assembly, two vimentin molecules interact in parallel to form a coiled coil. These coiled-coil dimers form anti-parallel, half-staggered tetramers. Typically, eight tetramers assemble laterally into ~65-nm long unit-length filaments (ULFs) [26], which then bind end-to-end to form non-polar filaments [27]. At least three major forms of vimentin can be seen in cells: particles (one or a few ULFs), squiggles (short filaments), and mature long filaments. Owing to their expression in mesenchymal and cancer cells, which tend to migrate, VIFs are thought to be more plastic than other types of IFs, and thus dynamic and suitable for cell migration and invasion. Indeed, VIFs are highly dynamic, constantly exchanging between vimentin filaments and disassembled subunits [28].

The balance between vimentin filaments and subunits is believed to be primarily regulated by serine phosphorylation [29, 30]. Vimentin has been shown to be phosphorylated by several serine/threonine kinases, and multiple serine phosphorylation sites have been identified [31–36]. During mitosis, VIFs are dramatically reorganized under the control of a series of phosphorylations to vimentin by Cdk1 and Plk1 from prometaphase to metaphase [37–39] and by Aurora-B and Rho-kinase from anaphase to the end of mitosis [40, 41]. Platelet-derived growth factor (PDGF) and epidermal growth factor (EGF) stimulate the spatial reorganization of VIFs in fibroblasts and endothelial cells;[42, 43] however, the underlying mechanism for this growth factor-induced reorganization of the VIFs remains obscure.

The cytoplasmic tyrosine kinase Src can be activated by a variety of cell surface receptors [44]. Src is known to regulate lamellipodia formation through Rac and Cdc42, and it promotes cell motility by stimulating focal adhesion dynamics [45]. In this study, we present a novel mechanism for regulating the dynamics of VIFs through Src-mediated tyrosine phosphorylation. We found that Src directly phosphorylates vimentin at Tyr117, which is crucial for the reorganization of VIFs in response to stimulation with PDGF and EGF. The nonreceptor protein tyrosine phosphatase SHP2 counteracts the effects of Src on VIF tyrosine phosphorylation and reorganization. Mutating vimentin at Tyr117 leads to abnormal VIF dynamics and adversely affects the lamellipodia formation and cell migration elicited by the growth factors. These results suggest that the proper control of VIF tyrosine phosphorylation and dynamics by Src and SHP2 is important for the cell migration elicited by growth factors.

## Results

### Src-mediated tyrosine phosphorylation of vimentin promotes the disassembly of VIFs in lamellipodia upon growth-factor stimulation

**Fig. 1:**
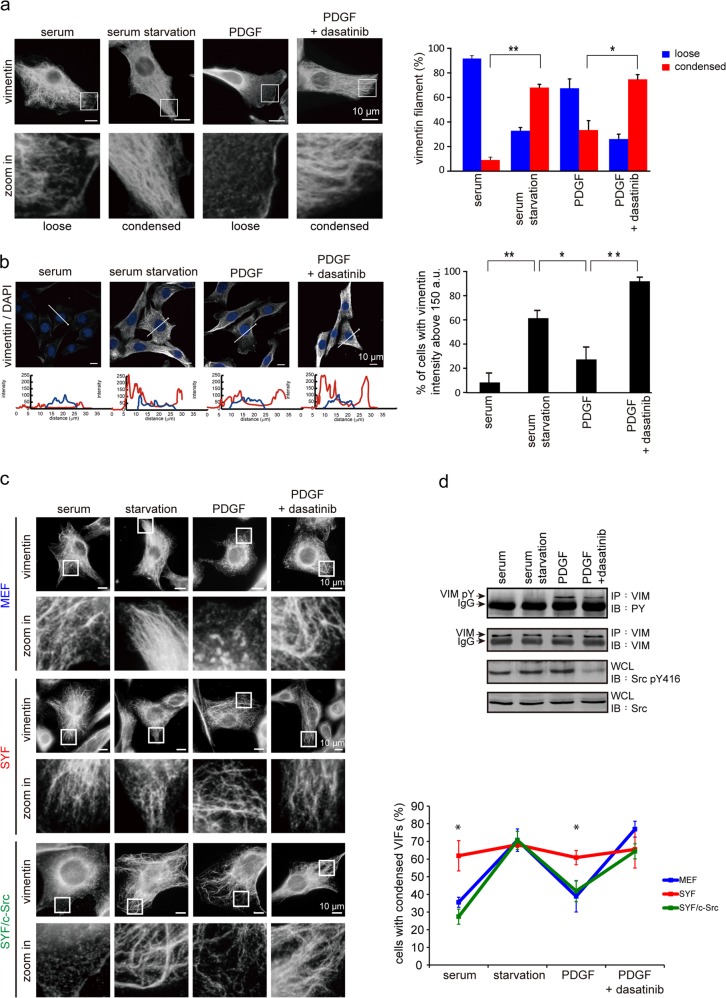
Src is important for the reorganization of VIFs upon growth-factor stimulation. **a** NIH3T3 cells were grown in medium supplemented with 10% serum or serum-free medium (starvation) for 48 h. The serum-starved cells were treated with 50 ng/mL PDGF in the presence or absence of 200 nM dasatinib for 6 h. The VIFs were visualized by immunofluorescence staining and their organization within a 10-μm range of the cell periphery was evaluated. Representative images taken with epifluorescence microscopy are shown, scale bars 10 μm. The loose VIFs are characterized by particle- and squiggle-type structures in the lamellipodia. The condensed VIFs are characterized by strong fluorescence intensity and extension to the cell periphery. The proportion of the total counted cells (*n* ≥ 300) with condensed or loose VIFs was determined. Values (means ± SD) are from three independent experiments. *P < 0.05, **P < 0.01. **b** NIH3T3 cells manipulated as described in **a** were fixed and stained for vimentin. Representative images taken with confocal fluorescence microscopy are shown, scale bars 10 μm. Graphs show the relative fluorescence intensity (F. I.) of the lines that were scanned by confocal microscopy. The fluorescence intensity was quantified with confocal fluorescence microscopy, and the proportion of the total counted cells (*n* ≥ 300) with a fluorescence intensity above 150 arbitrary units (a.u.) was determined. Values (means ± SD) are from three independent experiments. *P < 0.05, **P < 0.01. **c** MEF, SYF (src^-/-^, yes^-/-^, fyn^-/-^), and SYF/c-Src cells were incubated in the growth medium supplemented with 10% serum (growing) or serum-free medium (starvation) for 48 h. The serum-starved cells were then incubated with the medium supplemented with 10% serum in the presence or absence of 200 nM dasatinib for 6 h. The cells were fixed and stained for vimentin. Representative images from epifluorescence microscopy are shown. Note that the fluorescence intensity of the condensed VIFs is higher than that of the loose VIFs. The percentage of cells with condensed VIFs in the total counted cells was determined (*n* ≥ 300). Values (means ± SD) are from three independent experiments. *P < 0.05, **P < 0.01. **d** NIH3T3 cells as described in **a** were lysed in RIPA buffer. Vimentin was immunoprecipitated (IP) with anti-vimentin and the immunocomplexes were analyzed by immunoblotting (IB) with anti-phosphotyrosine (PY) or anti-vimentin. An equal amount of whole cell lysates was analyzed by immunoblotting with anti-Src and anti-Src pY416

To examine the role of Src in the reorganization of VIFs upon growth factor stimulation, NIH3T3 fibroblasts were serum-starved and then treated with PDGF in the presence or absence of the Src inhibitor dasatinib [46]. The VIFs were visualized by immunofluorescence staining and their organization within a 10-μm range of the cell periphery was evaluated. As shown in Fig. 1a, serum deprivation increased the assembly of VIFs, leading to their being condensed and extended to all parts of the cell periphery. When lamellipodia formation was induced by PDGF stimulation, the VIFs broke down into squiggles and particles at the cell edge, with their overall structure in these areas becoming much looser. In contrast, the Src inhibitor dasatinib antagonized PDGF’s effects on VIFs, and they remained condensed (Fig. 1a) with increased VIF fluorescence intensity as measured with confocal microscopy (Fig. 1b). The reorganization of VIFs was visualized in live NIH3T3 cells that stably expressed mCherry-vimentin with time-lapse microscopy (Supp. Fig. 1a). Indeed, PDGF promoted the dynamics and reorganization of VIFs along with the formation of lamellipodia, which was abolished by dasatinib (Supp. Fig. 1a).

To further examine the role of Src in the PDGF-induced VIF reorganization, mouse embryonic fibroblasts (MEFs) deficient in the Src family genes (SYF cells) were employed. In medium supplemented with 10% serum, the proportion of SYF cells exhibiting condensed VIFs was ~30% higher than their wild-type (WT) counterparts (Fig. 1c). Notably, the VIFs in the SYF cells remained condensed when stimulated with PDGF, and their responsiveness to PDGF was restored by expressing c-Src (Fig. 1c). The reorganization of VIFs was also examined in human cervical carcinoma HeLa cells that express both vimentin and keratin 18 (a type I cytokeratin). Upon EGF stimulation, the VIFs at the edge of the cell disassembled, while the keratin 18 filaments remained intact (Supp. Fig. 1b). Moreover, dasatinib suppressed the ability of EGF to induce the disassembly of VIFs (Supp. Fig. 1b). During growth-factor stimulation, the extent of VIF assembly was inversely correlated with the level of vimentin tyrosine phosphorylation (Fig. 1d and Supp. Fig. 1c). In contrast, EGF stimulation did not induce the tyrosine phosphorylation of keratin 18 in HeLa cells (Supp. Fig. 1c). Together, these results suggest that under growth-factor stimulation, the Src-mediated tyrosine phosphorylation of vimentin promotes VIF disassembly.

### SHP2 counteracts the effect of Src on VIFs

**Fig. 2:**
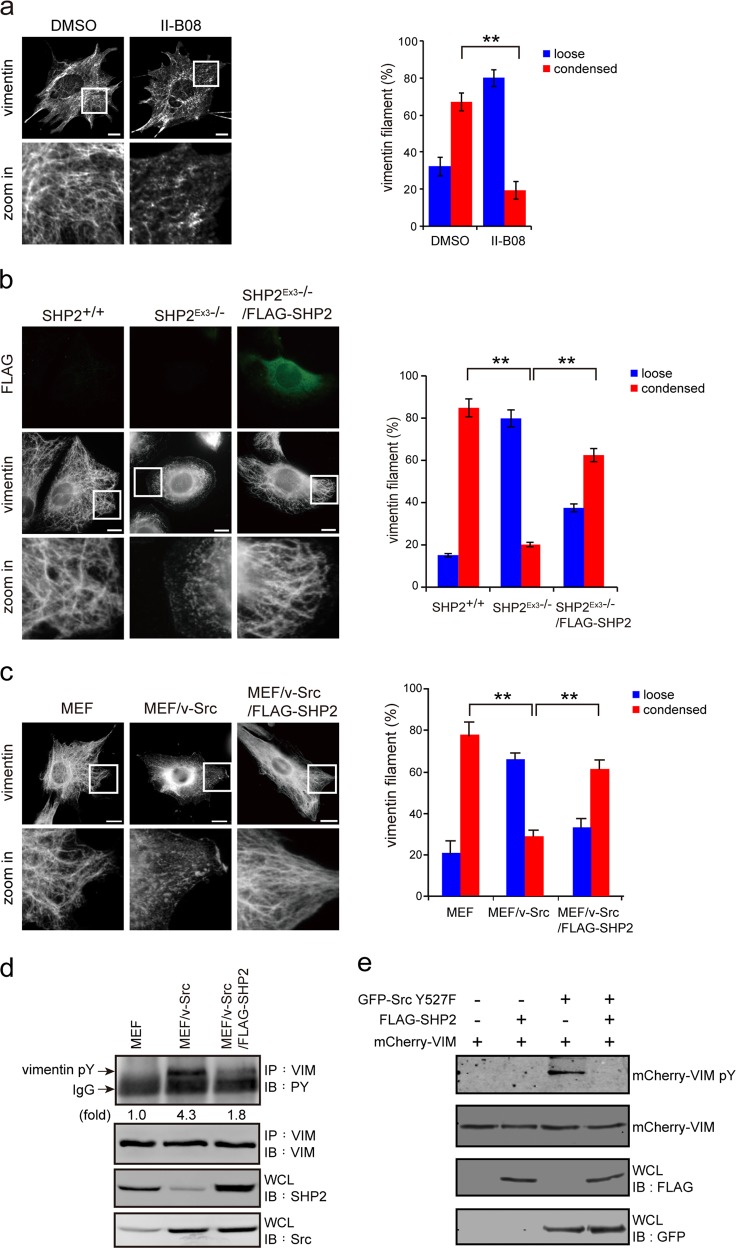
SHP2 counteracts the effect of Src on VIF tyrosine phosphorylation and organization. **a** MEFs were treated with the SHP2 inhibitor II-B08 (20 μM) for 6 h with the solvent dimethyl sulfoxide (DMSO) used as the control. The cells were then fixed and stained for vimentin. Representative images taken with epifluorescence microscopy are shown, scale bars 10 μm. The proportion of the total counted cells (*n* ≥ 300) with loose or condensed VIFs was determined. Values (means ± SD) are from three independent experiments. **P < 0.01. **b** MEFs with a deletion in exon 3 of the *shp2* gene (SHP2^Ex3-/-^), the wild type counterparts (SHP2^+/+^), and SHP2^Ex3-/-^ cells transiently expressing FLAG-SHP2 (SHP2^Ex3-/-^/FLAG-SHP2) were fixed and stained with anti-vimentin and anti-FLAG. Representative images taken with epifluorescence microscopy are shown. Scale bars 10 μm. The proportion of the total counted cells (*n* ≥ 300) with loose or condensed VIFs was determined. Values (means ± SD) are from three independent experiments. **P < 0.01. **c** MEFs, v-Src-transformed MEFs (MEF/v-Src), and v-Src-transformed MEFs stably expressing FLAG-SHP2 (v-Src/FLAG-SHP2) were fixed and stained for vimentin. Representative images taken with epifluorescence microscopy are shown. Scale bars 10 μm. The proportion of the total counted cells (*n* ≥ 300) with loose or condensed VIFs was determined. Values (means ± SD) are from three independent experiments. **P < 0.01. **d** The cells as described in **c** were lysed in RIPA buffer. Vimentin was immunoprecipitated (IP) with anti-vimentin and the immunocomplexes were analyzed by immunoblotting (IB) with anti-PY or anti-vimentin. An equal amount of whole cell lysates was analyzed by immunoblotting with anti-SHP2 and anti-Src. **e** mCherry-vimentin was transiently co-expressed with (+) or without (−) GFP-Src Y527F in HEK293 cells and then immunoprecipitated by anti-mCherry. The immunocomplexes were subjected to an in vitro phosphatase assay in the presence of purified FLAG-SHP2. The tyrosine phosphorylation of mCherry-vimentin was analyzed by immunoblotting with anti-phosphotyrosine

In our efforts to identify the protein tyrosine phosphatase that counteracts the effect of Src on VIFs, we found that the SHP2 inhibitor II-B08 [47] increased the proportion of MEFs with a loose VIF organization (Fig. 2a). To further examine the role of SHP2 in VIF organization, MEFs (SHP2^exon3-/-^) with a deletion in exon 3 of the *shp2* gene [48] were employed. The majority (~80%) of the SHP2^exon3-/-^ MEFs exhibited particle and squiggle VIFs, and ectopic expression of FLAG-SHP2 in the cells restored their condensed-network organization (Fig. 2b). Moreover, oncogenic vSrc induced the reorganization of VIFs from a condensed network to loose particles in MEFs. This was also reversed by the expression of FLAG-SHP2 (Fig. 2c), which reduced the vSrc-induced tyrosine phosphorylation of the VIFs (Fig. 2d). SHP2 was able to directly dephosphorylate vimentin that had been tyrosine phosphorylated by Src (Fig. 2e). These results indicate that SHP2 counteracts the effects of Src on VIF tyrosine phosphorylation and organization.

### Src directly phosphorylates vimentin at tyrosine 117

**Fig. 3:**
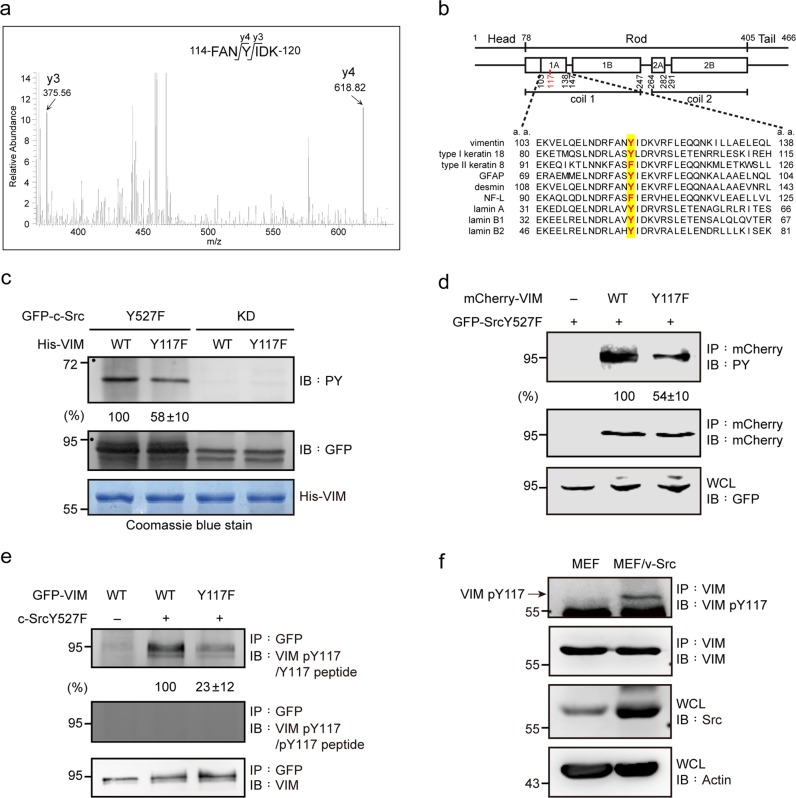
Src directly phosphorylates vimentin at Tyr117. **a** Mass spectrometry was used to analyze vimentin purified from v-Src-transformed MEFs. The mass spectrum indicates that vimentin Tyr117 is phosphorylated. **b** Tyr117 is located in the coil 1A region of vimentin, which is conserved in most, but not all, intermediate filaments. **c** Bacterially expressed His-tagged vimentin (His-VIM) and the Y117F mutant were purified and served as substrates for GFP-SrcY527F or the kinase-deficient (KD) mutant in vitro. The tyrosine phosphorylation of His-vimentin by GFP-SrcY527F was analyzed by immunoblotting with anti-PY. The amount of GFP-Src proteins used in the assay was analyzed by immunoblotting with anti-GFP. Purified His-vimentin proteins used in the assay were visualized by Coomassie blue stain. The tyrosine phosphorylation of His-vimentin was quantified and expressed as a percentage relative to the WT level. **d** mCherry-vimentin and the Y117F mutant were transiently co-expressed with GFP-cSrcY527F in HEK293 cells. mCherry-vimentin was immunoprecipitated with anti-mCherry and the immunocomplexes were analyzed by immunoblotting with anti-PY or anti-mCherry. The tyrosine phosphorylation of mCherry-vimentin was quantified and expressed as a percentage relative to the WT level. An equal amount of whole cell lysates was analyzed by immunoblotting with anti-GFP. **e** GFP-vimentin (GFP-VIM) and the Y117F mutant were immunoprecipitated by anti-GFP and the immunocomplexes were analyzed by immunoblotting with anti-GFP and anti-VIM pY117 in the presence of 5 μM Y117 phosphopeptide (pY117 peptide) or Y117 peptide (control). The tyrosine phosphorylation of GFP-vimentin at pY117 was quantified and expressed as a percentage relative to the WT level. **f** The Y117 phosphorylation of vimentin in MEFs and v-Src-transformed MEFs was analyzed. Vimentin was immunoprecipitated with anti-vimentin and the immunocomplexes were analyzed by immunoblotting with anti-VIM pY117. An equal amount of whole cell lysates was analyzed by immunoblotting with anti-Src and anti-actin

A mass spectrometry analysis revealed that vimentin was phosphorylated at Tyr117 in the vSrc-transformed MEFs, but not in the control MEFs (Fig. 3a). Tyr117 is conserved in most, but not all, intermediate filaments (Fig. 3b). The constitutively active Src Y527F phosphorylated vimentin both in vitro (Fig. 3c) and in intact cells (Fig. 3d), while its kinase-deficient mutant did not. The substitution of vimentin Tyr117 with Phe reduced Src tyrosine phosphorylation by ~50% (Fig. 3c, d). To facilitate the detection of Tyr117-phosphorylated vimentin, an antibody (anti-VIM pY117) specific to phosphorylated Tyr117 was generated that recognized Src-phosphorylated vimentin, but not the Y117F mutant (Fig. 3e). The specificity of this antibody was confirmed by successfully blocking it with a phosphopeptide corresponding to the Tyr117 flanking sequences (Fig. 3e). This phospho-specific antibody demonstrated that Src directly phosphorylates Tyr117 in endogenous vimentin (Fig. 3f). Vimentin was previously reported to be phosphorylated at Tyr53 and Tyr61 in cancer cells [49]. In this study, we demonstrated that Tyr53 and Tyr61 of vimentin are likely to be phosphorylation sites for Src (Supp. Fig. 2). Triple mutations at Tyr53, Tyr61, and Tyr117 abolished the tyrosine phosphorylation of vimentin by Src (Supp. Fig. 2).

### Phosphorylation of vimentin at Tyr117 may prevent its assembly into filaments

**Fig. 4:**
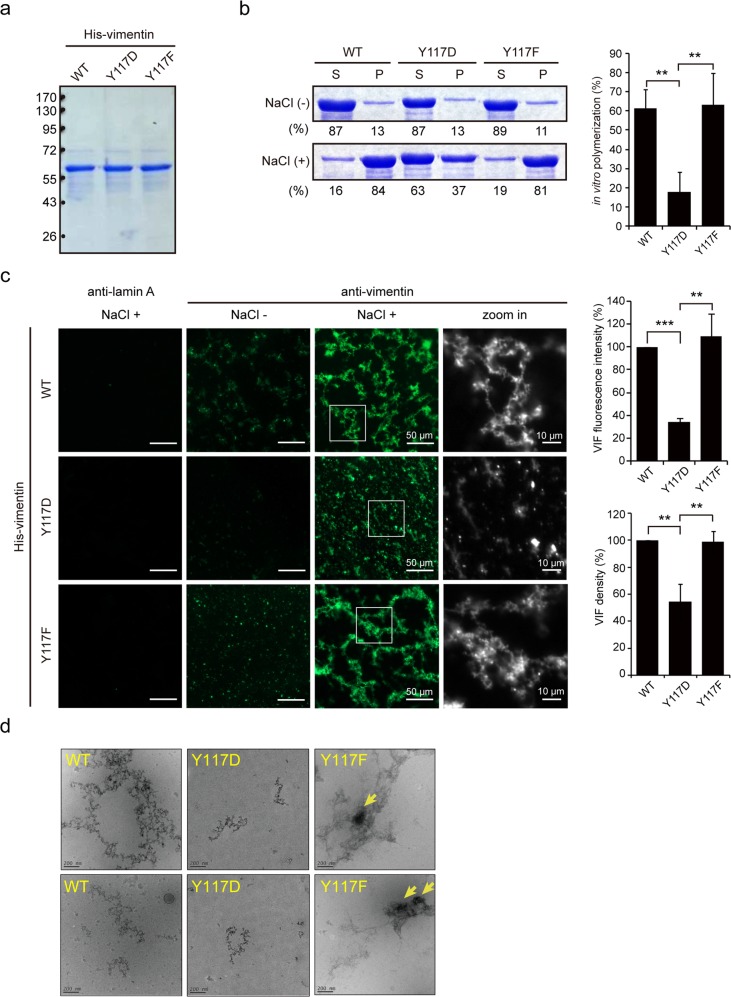
Substitution of vimentin Tyr117 with Asp prevents its polymerization in vitro. **a** Purified His-vimentin proteins were stored in a buffer without sodium chloride, fractionated by SDS-PAGE, and visualized by Coomassie blue stain. **b** The in vitro polymerization of His-vimentin was performed by the addition of 150 mM sodium chloride (NaCl) and incubation at 30 °C for 30 min. The proteins were then centrifuged at 100,000 × g at 4 °C for 20 min. The supernatants were collected and the pellets were solubilized in vimentin extraction buffer containing 7 M urea. Equal proportions of His-vimentin in the supernatant (S) and pellet (P) fractions were fractionated by SDS-PAGE and visualized by Coomassie blue stain. The ratio of His-vimentin in the supernatant and pellet fractions was measured with ImageJ software. The in vitro polymerization of His-vimentin is expressed as the net percentage of His-vimentin in the pellet with and without NaCl. Values (means ± SD) are from three independent experiments. **P < 0.01. **c** His-vimentin proteins were polymerized in the presence or absence of 150 mM NaCl at 30 °C for 30 min and visualized by immunofluorescence staining with anti-vimentin or anti-lamin (control). Images were acquired using a Zeiss ApoTome2 microscope imaging system. Scale bars 50 μm. The fluorescence intensity of VIFs per 400 µm^2^ was measured with the microscope imaging system (*n* ≥ 30). The VIF density was measured with Photoshop S6 (*n* ≥ 30). Data are expressed as a percentage relative to the WT level. Values (means ± SD) are from three independent experiments. **P < 0.01, ***P < 0.001. **d** The polymerized His-vimentin proteins were visualized by cryo-electron microscopy. Representative images are shown. Arrow indicates the aggregates formed by the His-VIM Y117F mutant. Scale bars 200 nm

The Tyr117 in vimentin is located in the coil 1A region and resides at the dimeric coiled-coil interface [50]. We hypothesized that once vimentin is phosphorylated at Tyr117, the phosphate group at that location would be too large to be accommodated at the interface and would electrostatically repel another vimentin from dimerization. To examine this possibility, purified His-tagged vimentin (His-vimentin) (Fig. 4a) was polymerized in vitro in the presence of 150 mM sodium chloride and then precipitated by centrifugation. A large portion of the phosphomimetic mutant Y117D was retained in the supernatant, whereas the majority of the WT and Y117F mutant were present in the pellet (Fig. 4b). The polymerized His-vimentin filaments were further visualized by immunofluorescence staining using anti-vimentin (Fig. 4c) and cryo-electron microscopy (Fig. 4d). The Y117D mutant was less able to form filaments in comparison to the WT and Y117F mutant (Fig. 4c). The Y117F mutant also appeared to form more aggregates than the WT (Fig. 4d). Moreover, mCherry-vimentin and the Tyr117 mutants were transiently expressed in HEK293 cells, and their solubility in 1% NP40 lysis buffer was examined. Whereas mCherry-vimentin and the Y117F mutant were insoluble in 1% NP40 and precipitated in the pellets, most of the Y117D mutant was soluble and remained in the supernatant (Supp. Fig. 3). Together, these data suggest that the phosphorylation of vimentin at Tyr117 may prevent its assembly in vitro.

### Phosphorylation of vimentin at Tyr117 is important for the growth factor-induced reorganization of VIFs in intact cells

**Fig. 5:**
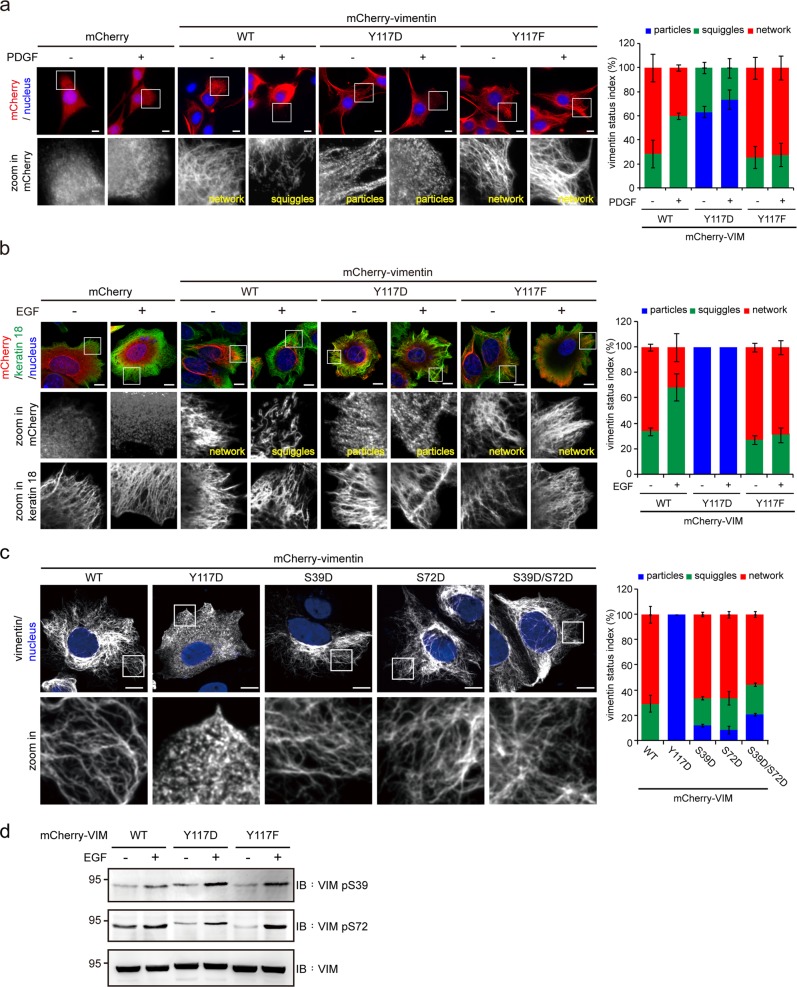
Phosphorylation of vimentin at Tyr117 is important for the growth factor-induced reorganization of VIFs in intact cells. **a** NIH3T3 cells stably expressing mCherry-vimentin-WT, Y117D, or Y117F were serum-starved for 24 h and then treated with (+) or without (−) 50 ng/mL PDGF for 6 h. The cells were then fixed and stained for mCherry. The organization of the mCherry-vimentin was visualized with a Zeiss ApoTome2 microscope imaging system. Scale bars 10 μm. The proportion of cells with particle-, squiggle-, or network-type VIFs in the total counted cells (*n* ≥ 300) was determined. Values (means ± SD) are from three independent experiments. **b** mCherry-vimentin WT, Y117D, and Y117F were transiently expressed in MCF7 cells that do not express endogenous vimentin. The cells were serum-starved for 24 h and then treated with (+) or without (−) 200 ng/mL EGF for 6 h. The cells were fixed and stained for mCherry and keratin 18. The organization of mCherry-vimentin and keratin 18 was visualized with Zeiss ApoTome2 microscope imaging system. Scale bars 10 μm. The proportion of cells with particle-, squiggle-, or network-type VIFs in the total counted cells (*n* ≥ 300) was determined. Values (means ± SD) are from three independent experiments. **c** mCherry-vimentin WT, Y117D, S39D, S72D, and S39D/S72D (double mutant) were transiently expressed in MCF7 cells, serum-starved for 24 h, and then visualized with epifluorescence microscopy. Scale bars 10 μm. The proportion of cells with particle-, squiggle-, or network-type VIFs in the total counted cells (*n* ≥ 300) was determined. Values (means ± SD) are from three independent experiments. **d** HeLa cells were serum-starved for 24 h, treated with (+) or without (−) 200 ng/mL EGF for 6 h, and then lysed with RIPA buffer. An equal amount of whole cell lysates was analyzed by immunoblotting with anti-vimentin, anti-vimentin pS39, and anti-vimentin pS72

The organization of mCherry-vimentin and the Tyr117 mutants was visualized in NIH3T3 cells (Fig. 5a). We found that mCherry-vimentin and the Y117F mutant assembled into condensed networks in most (~75%) cells, whereas the Y117D mutant was primarily (~65%) present as particles (Fig. 5a). When stimulated with PDGF, the proportion of mCherry-vimentin WT squiggles increased, whereas the Y117F mutant did not respond (Fig. 5a). To avoid the potential effects of endogenous vimentin on the assembly of mCherry-vimentin in the NIH3T3 cells, the organization of mCherry-vimentin and the mutants was also visualized in human breast cancer MCF7 cells that do not express endogenous vimentin (Fig. 5b). In these cells, the Y117D mutant exhibited as particles in all cells. When stimulated with EGF, the network formed by the mCherry-vimentin WT disassembled into squiggles (Fig. 5b), whereas the network formed by the Y117F mutant was unaffected (Fig. 5b). These results suggest that the phosphorylation of vimentin at Tyr117 is important for the reorganization of VIFs upon growth-factor stimulation.

The phosphorylation of vimentin at Ser39 and Ser72 by ROCK and PAK has been reported to facilitate the disassembly of VIFs [23, 40]. However, in contrast to the mCherry-vimentin Y117D mutant that formed only particle-type structures in serum-starved MCF7 cells, the S39D, S72D, and S39D/S72D mutants were able to form networks (Fig. 5c). In addition, upon EGF stimulation, the Y117F mutant that formed the VIF networks in MCF7 cells was phosphorylated at Ser39 and Ser73 (Fig. 5d). Together, these results suggest that in response to growth-factor stimulation, phosphorylation at vimentin Tyr117 may have a more profound effect on the reorganization of VIFs than does serine phosphorylation.

### Phosphorylation of vimentin at Tyr117 is crucial for VIF dynamics

**Fig. 6:**
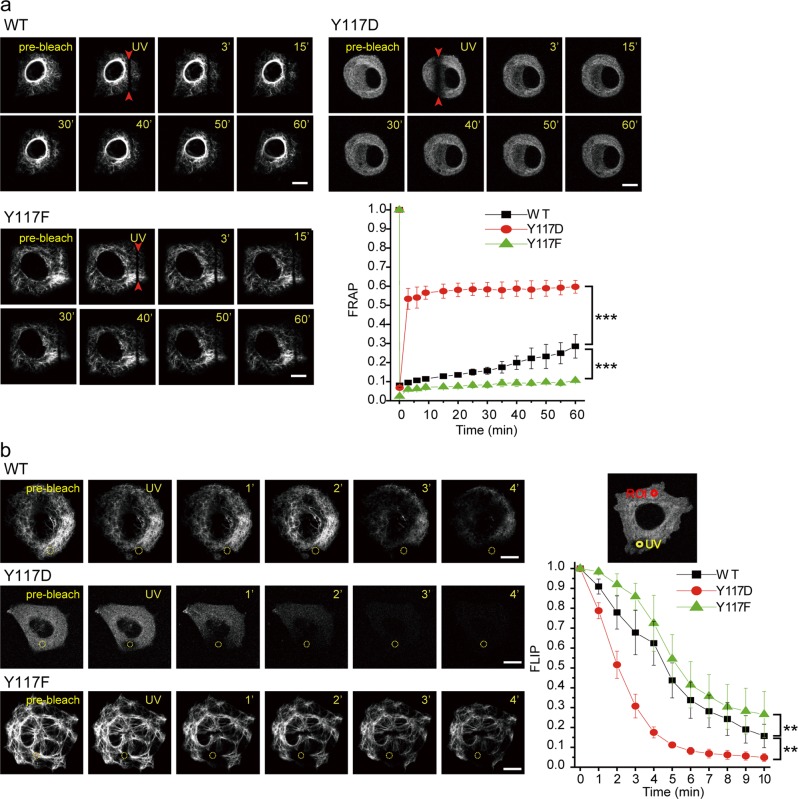
Phosphorylation of vimentin at Tyr117 is critical for VIF dynamics. **a** A fluorescence recovery after photobleaching (FRAP) analysis was performed in MCF cells transiently expressing mCherry-vimentin-WT, Y117D, or Y117F. The selected regions (indicated by red arrowheads) were photobleached once by a 405-nm laser for 1 s. Representative images from confocal fluorescence microscopy (543 nm excitation) before bleaching (pre-bleach) and at different time points after bleaching are shown. Scale bars 10 μm. Fluorescence at the photobleached regions (indicated by red arrowheads) was measured and FRAP was calculated as a ratio to the initial fluorescence. Values (means ± SD) are from at least three independent experiments. Two-way ANOVAs with Tukey post-hoc tests were used for the comparisons of WT vs. Y117D and WT vs. Y117F at each time point. ***P < 0.001. **b** A fluorescence loss in photobleaching (FLIP) analysis was performed in MCF7 cells transiently expressing mCherry-vimentin WT, Y117D, or Y117F. The selected regions (yellow circle, 3 μm diameter) were photobleached by a 405-nm laser for 10 min. Representative images from confocal fluorescence microscopy (543 nm excitation) before bleaching (pre-bleach) and at different time points after bleaching are shown. Scale bars 10 μm. Fluorescence at the regions of interest (ROI red circles, 3 μm diameter) was measured, and FLIP was calculated as a ratio to the initial fluorescence. Values (means ± SD) are from at least four independent experiments. Two-way ANOVAs with Tukey post-hoc tests were used for the comparisons of WT vs. Y117D and WT vs. Y117F at each time point. **P < 0.01

To examine the dynamics of mCherry-vimentin in live cells, two fluorescence microscopy techniques—fluorescence recovery after photobleaching (FRAP) and fluorescence loss in photobleaching (FLIP)—were performed in MCF7 cells transiently expressing mCherry-vimentin. The Y117D mutant was diffusible and very dynamic, recovering ~55% of the photobleached zone within 3 min after photobleaching (Fig. 6a) and exhibiting much faster FLIP than the WT and Y117F mutant (Fig. 6b). One hour after photobleaching, the mCherry-vimentin WT and Y117F mutant had recovered ~30% and ~10% of the photobleached zone, respectively (Fig. 6a), suggesting that the Y117F mutant is less dynamic than the WT. Accordingly, the FLIP of the Y117F mutant was slower than that of WT (Fig. 6b). Moreover, the dynamics of mCherry-vimentin in live MCF7 cells was affected by the SHP2 inhibitor II-B08 (Supp. Fig. 4), which increased the proportion of cells with loosely organized mCherry-vimentin (Supp. Fig. 4a). As measured by FRAP and FLIP, the dynamics of mCherry-vimentin was increased by II-B08 (Supp. Fig. 4b and 4c). These results suggest that the coordinate regulation of Tyr117 phosphorylation by Src and SHP2 may be critical for VIF dynamics.

### Proper VIF dynamics are important for cell migration through the facilitation of lamellipodia formation

**Fig. 7:**
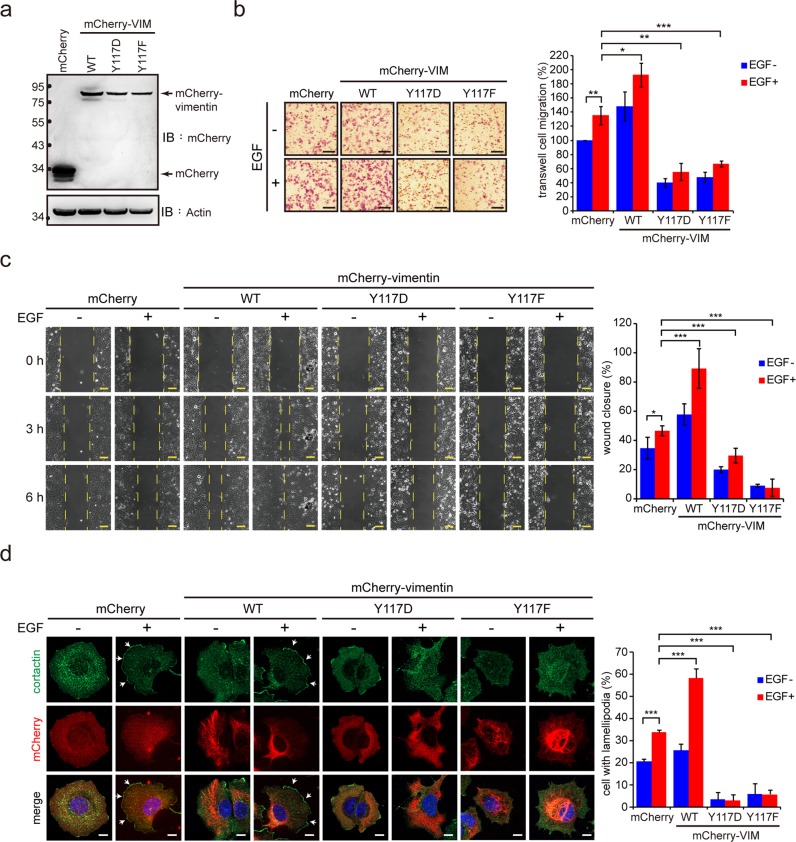
Proper VIF dynamics are important for lamellipodia formation and cell migration. **a** Equal amounts of whole cell lysates from MCF7 cells stably expressing mCherry-VIM WT, Y117D, and Y117F or mCherry alone (control) were analyzed by immunoblotting with anti-mCherry or anti-actin. **b** MCF7 cells (5 × 10^3^) were subjected to a trans-well cell migration assay in the presence (+) or absence (−) 200 ng/mL EGF for 24 h. The cells that migrated to the lower chamber were fixed, stained, and counted. Representative micrographs are shown, scale bars 100 μm. Data are expressed as a proportion of the control cell levels in the absence of EGF. Values (means ± SD) are from three independent experiments. *P < 0.05, **P < 0.01, ***P < 0.001. **c** The migration of MCF7 cells was analyzed by a wound-healing assay with an ibidi Culture-Insert. The cells (3 × 10^4^) were grown as a monolayer and serum-starved for 24 h. A 500 ± 50 µm cell-free gap was generated and the cells were treated with (+) or without (−) 200 ng/mL EGF. The closure of the gap was visualized with time-lapse microscopy with micrographs taken every 5 min for 6 h. Representative micrographs at 0, 3, and 6 h are shown. Scale bars 100 μm. Data are expressed as a percentage of the gap closure at 6 h. Values (means ± SD) are from three independent experiments. *P < 0.05, ***P < 0.001. **d** MCF7 cells were serum-starved for 24 h and then treated with (+) or without (−) 200 ng/mL EGF for 1.5 h. The cells were fixed and stained for cortactin, which serves as a marker for lamellipodia. Images were acquired with a Zeiss ApoTome2 microscope imaging system. Arrows indicate lamellipodia. Scale bars 10 μm. The proportion of cells with lamellipodia relative to the total counted cells (*n* ≥ 300) was determined. Values (means ± SD) are from three independent experiments. ***P < 0.001

To examine the effect of VIF dynamics on cell migration, mCherry-vimentin and the Tyr117 mutants were stably expressed in MCF7 (Fig. 7) and NIH3T3 cells (Supp. Fig. 5). The expression of mCherry-vimentin by itself promoted cell migration to some extent, and cell migration was further promoted when the MCF7 and NIH3T3 cells were stimulated with EGF (Fig. 7a–c) and PDGF (Supp. Fig. 5), respectively. In contrast, the Y117D and Y117F mutants behaved in a dominant-negative manner and suppressed cell migration in both the MCF7 and NIH3T3 cells. Both the mutants also suppressed v-Src-promoted cell migration (Supp. Fig 6a and 6b). However, the Y117F mutant, but not the Y117D mutant, partially inhibited the anchorage-independent growth of v-Src-transformed cells (Supp. Fig. 6c). During cell migration, a cell forms lamellipodia at its leading edge, and the membrane extension (the lamellipodia) can be identified by the cortactin distribution [51]. We found that in MCF7 cells stimulated by EGF, the expression of mCherry-vimentin promoted lamellipodia formation (Fig. 7d). In contrast, the Y117D and Y117F mutants exerted a dominant-negative effect on lamellipodia formation (Fig. 7d).

### Upon growth factor stimulation, proper VIF dynamics are important for the translocation of Vav2 to the plasma membrane and Rac activation

**Fig. 8:**
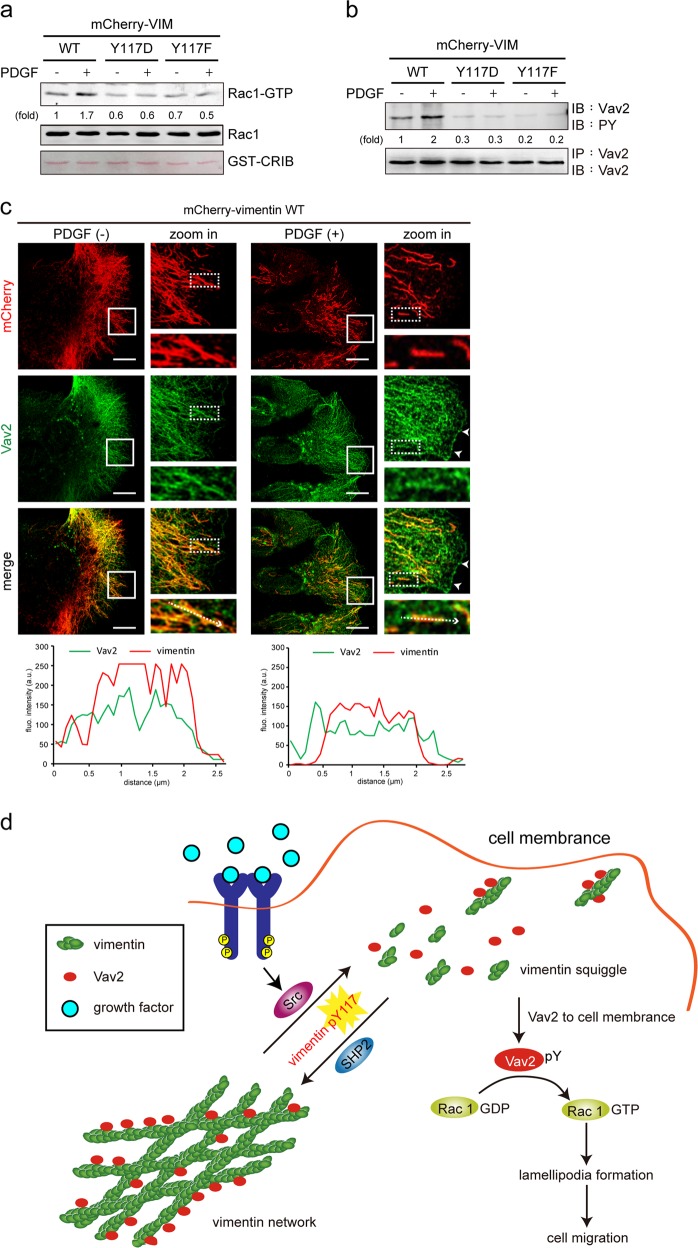
Proper VIF dynamics are important for the translocation of Vav2 to the plasma membrane and Rac1 activation. **a** NIH3T3 cells stably expressing mCherry-vimentin, Y117D, or Y117F were serum-starved for 24 h and then treated with (+) or without (−) 50 ng/mL PDGF for 6 h. The GTP-bound Rac1 was pulled down by GST-PAK-CRIB and detected by anti-Rac1. Total Rac1 proteins in the cell lysates were analyzed by immunoblotting with ant-Rac. GST-PAK-CRIB was visualized by Ponceau S Stain. **b** Vav2 was immunoprecipitated (IP) with anti-Vav2, and the immunocomplexes were analyzed by immunoblotting (IB) with anti-PY or anti-Vav2. **c** NIH3T3 cells stably expressing mCherry-vimentin were serum-starved for 24 h and treated with (+) or without (−) 50 ng/mL PDGF for 6 h. The cells were fixed and stained with anti-mCherry and anti-Vav2. Representative images were taken with confocal microscopy. Arrowheads indicate the membrane localization of Vav2. Scale bars 10 μm. Graphs show the relative fluorescence intensity of the lines that were scanned by confocal microscopy. **d** Model of VIF dynamics regulated by Src and SHP2 and their link to lamellipodia formation through the Vav2-Rac signaling pathway

Lamellipodia formation is known to be regulated by the small GTPase Rac [21]. We found that the expression of mCherry-vimentin promoted Rac activation upon PDGF stimulation, whereas the Y117D and Y117F mutants suppressed PDGF-induced Rac activation (Fig. 8a). The activation of Rac was correlated with an increase in the tyrosine phosphorylation of Vav2 (Fig. 8b), a guanine-nucleotide exchange factor (GEF) [52]; the tyrosine phosphorylation of Vav2 is an indicator of its activation [53]. Surprisingly, we found that Vav2 was largely associated with VIFs prior to PDGF stimulation (Fig. 8c), after which, the disassembly of VIFs was accompanied by the recruitment of Vav2 to the plasma membrane (Fig. 8c). These results suggest that upon growth-factor stimulation, the phosphorylation of vimentin Tyr117 to maintain proper VIF dynamics is important for the recruitment of Vav2 to the plasma membrane and subsequent Rac activation.

## Discussion

The assembly state of VIFs are known to be regulated by serine phosphorylation. Although the tyrosine phosphorylation of vimentin was reported two decades ago [43], the underlying mechanism and functional consequences remained unclear. In this study, we demonstrated that Src and SHP2 coordinately regulate VIF tyrosine phosphorylation and organization upon growth-factor stimulation (Figs. 1 and 2). Tyr117 was identified as the major vimentin phosphorylation site for Src (Fig. 3), and this phosphorylation appears to prevent the assembly of vimentin into filaments both in vitro (Fig. 4) and in intact cells (Fig. 5). Our results suggest that the coordinate regulation of vimentin Tyr117 phosphorylation by Src and SHP2 is importat for VIF dynamics (Fig. 6), which appears to be essential for the activation of the Vav2-Rac1 signaling pathway that leads to lamellipodia formation and cell migration (Fig. 7). In this study, we found that the activation of Vav2 and Rac1 by PDGF was inhibited by the Y117D and Y117F mutants (Fig. 8a, b), suggesting that proper VIF dynamics are important for Vav2 and Rac1 activation. Surprisingly, we found that Vav2 was primarily associated with VIFs in serum-starved cells (Fig. 8c). Upon PDGF stimulation, the VIFs were converted into squiggles and particles, accompanied by the translocation of Vav2 to the plasma membrane, which is necessary for the activation of Rac1. Given that vimentin precursors move along microtubules via kinesin and dynein motors [54], which drive the front-rear polarity of intermediate filaments in motile cells [55], our results raise the possibility that upon growth-factor stimulation, vimentin squiggles and/or particles may carry and transport Vav2 to the plasma membrane along microtubule tracks (Fig. 8d).

In this study, we have proposed that upon growth-factor stimulation, proper VIF dynamics are important for lamellipodia formation and cell migration. Previous studies have shown that vimentin can promote cell migration through its effects on cell adhesion and contractility. Vimentin has been found to localize in focal adhesions and play an important role in regulating their size and turnover [16, 56]. It also forms complexes with Vav2 in the focal adhesions and is important for Vav2 and focal adhesion kinase (FAK) activity [57]. A lack of vimentin has been shown to attenuate the activity of FAK and upregulate RhoA activity through a FAK-dependent compensatory feedback loop [58]. In addition, vimentin filaments were shown to negatively regulate stress fiber assembly and contractility via the suppression of the microtubule-associated RhoA exchange factor GEF-H1 [59]. Vimentin depletion induces extensive stress fiber formation through the activation of GEF-H1, and thereby RhoA, leading to an inhibition of cell motility [59]. Vimentin can also affect cell motility through integrin recycling in a PKCε-dependent manner [15]. Taken together, these studies and ours suggest that vimentin may regulate cell motility through different routes depending on the cell type and extracellular stimuli.

A recent study showed that when a photoactivatable Rac1 is locally activated in serum-starved cells, VIFs are converted into squiggles and particles in the proximal region of the activated Rac1 [20], which suggests that Rac1 activation may precede VIF disassembly. In this scenario, PAK would act downstream of Rac1 to phosphorylate and induce the disassembly of VIFs. However, the results in this study suggest that the proper disassembly and dynamics of VIFs are important for the activation of the Vav2-Rac1 pathway upon growth-factor stimulation. This is consistent with the observation that the microinjection of a mimetic peptide into serum-starved cells, which causes the disassembly of VIFs into ULFs, is sufficient to locally induce the disassembly of VIFs and initiate lamellipodia formation [20]. In fact, Vav2 has been suggested to mediate the effect of VIFs on Rac1 activation [57]. However, the underlying mechanism remains unclear.

In this study, we identified vimentin Tyr117 as the major phosphorylation site for Src (Fig. 4). This residue was first noticed because its substitution with Leu stabilizes the dimeric form of the vimentin coil 1A peptide comprised of residues 103–138 (ref. 50.). The x-ray crystallography of the coil 1A^Y117L^ dimer revealed that residue 117 resides at the dimeric coiled-coil interface [50]. When this mutation was introduced into full-length vimentin, the Y117L variant formed ULF particles, but they did not anneal longitudinally and thus could not form VIFs [50]. As a result, vimentin^Y117L^ has been used as a tool to study the transport of ULF particles in cells [60]. In this study, we found that vimentin^Y117D^ also forms ULF-like particles both in vitro and in cells. However, it is not known whether the properties of the ULFs formed by vimentin^Y117D^ are the same as those formed by vimentin^Y117L^. One question is how the phosphate group at Tyr117 affects the assembly of vimentin into filaments. A recent study [61] indicates that an ATP-dependent cofactor (e.g., a kinase or an ATP-dependent chaperone) is required for the maintence of vimentin at the ULF level in equilibrium with soluble vimentin tetramers. Our results suggest that Src may be the ATP-dependent co-factor that induces the dissociation of ULF tetramers and prevents them from annealing longitudinally.

The non-α-helical NH2-terminal head domain of vimentin contains several known serine phosphorylation sites on vimentin. However, it is not clear whether the serine phosphorylation and Tyr117 phosphorylation are reciprocally regulated. Upon EGF stimulation, the Y117F mutant can be detected to be phosphorylated at Ser39 and Ser72 (Fig. 5d), indicating that Tyr117 phosphorylation is not required for the serine phosphorylation in the head domain. However, it is not clear whether the serine phosphorylation in the NH2-terminal head domain is required for Tyr117 phosphorylation. It is possible that the serine phosphorylation in the head domain may unwind the coiled-coil structure of the VIFs, thereby allowing Src to access the Tyr117 for phosphorylation.

In this study, we found that EGF induced the tyrosine phosphorylation of vimentin, but not keratin 18 (Supp. Fig. 1c) in HeLa cells. Nevertheless, tyrosine phosphorylation has been reported in some types of keratins. For example, keratin 19, a type I intermediate filament protein, was reported to be phosphorylated at Tyr391 in the tail domain in the presence of Src or when treated with pervanadate [62], but the functional significance of this remains unknown. In addition, keratin 8, a type II intermediate filament protein, was shown to be phosphorylated on Tyr267 in the rod domain [63]. Although the tyrosine kinase responsible for the Tyr267 phosphorylation is uncertain, this phosphorylation site is targeted by the phosphatase PTP1B [63]. Importantly, Tyr267 phosphorylation decreases the solubility of keratin 8 filaments [63]. Therefore, the functional consequence of Src-mediated tyrosine phosphorylation on keratin filaments may be different from that on vimentin filaments. Moreover, Src was shown to directly interact with keratin 6 (a type II intermediate filament protein), which suppresses the kinase activity of Src and cell migration of skin keratinocytes during wound repair [64]. The potential impact on Src activity by the different VIF configurations remains to be examined.

Src can be activated by a variety of cell surface receptors [44], and our study provides an explanation for how vimentin plasticity is regulated in response to their extracellular cues. In addition to fibroblasts and cancer cells, vimentin is also expressed in leukocytes and endothelial cells. Given that Src family members play important roles in the physiological functions of those cells, Src (or its family members) may also be involved in vimentin filament regulation when the cells are exposed to extracellular stimuli such as cytokine stimulation in leukocytes or fluid shear stress in endothelial cells. Moreover, because Tyr117 and its flanking sequences are conserved in some types of intermediate filament proteins, such as lamin and glial fibrillary acidic protein, our study raises the intriguing possibility that Src-mediated phosphorylation may represent a general mechanism for regulating the plasticity of intermediate filaments. Further studies will be necessary to confirm this possibility.

## Materials and methods

### Materials

The rabbit polyclonal anti-vimentin (for immunoprecipitation) and anti-vimentin pY117 antibodies were generated using purified His-vimentin and a synthesized phospho-peptide (ELNDRFANpYIDKVR-C; pY117 peptide), respectively, as the antigens through a custom antibody production service provided by GeneTex, Inc. (Hsinchu, Taiwan). Mouse ascites containing monoclonal anti-Src antibody (2–17) produced by a hybridoma (CRL2651) were prepared in our laboratory. The mouse monoclonal anti-vimentin (clone 13.2 and V9), anti-keratin 18 (CY-90), and anti-actin (AC-15) antibodies, rabbit polyclonal anti-FLAG antibody (#F3165), human collagen I, and protein A–Sepharose beads were purchased from Sigma-Aldrich. The rabbit monoclonal anti-vimentin pS72 (EP1070Y), and rabbit polyclonal anti-mCherry (ab183628) antibodies were purchased from Abcam. The rabbit monoclonal anti-Src pY416 (D49G4) antibody and rabbit polyclonal anti-vimentin pS39 (#13614) antibody were purchased from Cell Signaling Technology. The monoclonal anti-phosphotyrosine (anti-PY; 4G10) antibody, the SHP2 inhibitor II-B08, and fibronectin were purchased from Merck Millipore. The mouse monoclonal anti-Rac1 (clone 102) antibody was purchased from BD Transduction Laboratories. The mouse monoclonal anti-GFP (clones 13.1 and 7.1) antibody was purchased from Roche. The mouse monoclonal anti-GFP (B-2) and anti-Vav2 (F-6) antibodies, and rabbit polyclonal anti-Vav2 (H-200) and anti-vimentin (C-20) antibodies were purchased from Santa Cruz Biotechnology. The HRP-conjugated goat anti-mouse and goat anti-rabbit antibodies and rabbit anti-mouse IgG were purchased from Jackson ImmunoResearch Laboratories. Dulbecco’s modified Eagle’s medium (DMEM), Zeocin, Lipofectamine, Alexa Fluor 488- and Alexa Fluor 546-conjugated secondary antibodies were purchased from Invitrogen Life Technologies. Glutathione Sepharose was purchased from GE Healthcare Life Sciences. PDGF was purchased from PeproTech (Rocky Hill, NJ), and EGF was purchased from R&D Systems (Minneapolis. MN). The Src inhibitor dasatinib was purchased from BioVision (Milpitas, CA). Dialysis tubing (15.5 mm wet diameter) was purchased from BioDesign (Carmel, New York).

### Plasmids

The plasmids pEVX-cSrc WT and its mutants (Y527F and K295R) were described previously [65]. For GFP-Src, the cDNAs of cSrc and the mutants were subcloned into the pEGFP-N3 plasmid using the EcoRI and BamHI sites. The plasmids pFLAG-CMV2-SHP2 and pLKOAS2.zeo-FLAG-SHP2 were described previously [66]. The plasmids pEGFP-N1-vimentin and pmCherry-vimentin series (WT, S39D, and S72D) were described previously [67]. For His-tagged vimentin, vimentin cDNA was cloned into the pET-21-b(+) plasmid with the NheI and EcoRI sites. All mutagenesis was performed using the QuikChange site-directed mutagenesis kit (Agilent Technologies) and the desired mutations were confirmed by dideoxy DNA sequencing.

### Cell culture and transfection

NIH3T3 cells, MEFs, v-Src-transformed MEFs [67] and v-Src-transformed MEFs stably expressing FLAG-SHP2 [68] were described previously. v-Src-transformed NIH3T3 cells were described previously [69]. MEF/SHP2^EX3-/-^ cells were kindly provided by Dr. Gen-Sheng Feng (University of California San Diego, USA) and described previously [48]. SYF (src^-/-^ yes^-/-^ fyn^-/-^) cells and SYF/c-Src cells were described previously [70]. The HEK293, MCF7, and HeLa cells were purchased from the American Type Culture Collection. HEK293, NIH3T3, NIH3T3/v-Src, MCF7, HeLa, MEF, MEF/v-Src, and SYF cells were maintained in DMEM supplemented with 10% fetal bovine serum (Hyclone). To generate MCF7 cells stably expressing mCherry-vimentin, the MCF7 cells were transfected with pmCherry-vimentin or its mutants using Lipofectamine. The cells were then selected in medium containing 0.5 mg/mL neomycin two days after transfection. Ten days later, the neomycin-resistant cells were pooled and analyzed for exogenous vimentin expression by immunoblotting with anti-mCherry. To generate NIH3T3 cells stably expressing mCherry-vimentin, the NIH3T3 cells were infected with lentiviruses encoding mCherry-vimentin or its mutants for 24 h and subsequently selected in medium containing 1 mg/mL neomycin. Ten days later, the neomycin-resistant cells were analyzed for mCherry-vimentin by immunoblotting.

### Lentivirus production and infection

The lentiviral expression system was provided by the National RNAi Core Facility, Academia Sinica, Taiwan. For mCherry-vimentin WT, Y117D, and Y117F expression, the respective cDNA were amplified by polymerase chain reaction and subcloned in frame to the NheI and EcoRI site of the pLAS3w-pNeo vector. To produce the lentiviruses, HEK293T cells were co-transfected with 2.25 μg pCMV-ΔR8.91, 0.25 μg pMD.G, and 2.5 μg pLKO-AS2-zeo-FLAG-SHP2 (or pLAS3w-pNeo-mCherry-vimentin) using Lipofectamine. After 3 days, the medium containing the viral particles was collected and stored at −80 °C. The cells were infected by lentiviruses capable of expressing FLAG-SHP2 or mCherry-vimentin and selected in the medium containing Zeomycin (100–500 µg/mL) or neomycin (0.5–1.0 mg/mL), respectively.

### Immunoblotting and immunoprecipitation

Cells were lysed in either 1% NP-40 lysis buffer (1% Nonidet P-40, 20 mM Tris-HCl, pH 8.0, 137 mM NaCl, 10% glycerol, and 1 mM Na_3_VO_4_) or RIPA lysis buffer (1% Nonidet P-40, 50 mM Tris-HCl, pH 7.4, 1% Na-deoxycholate, 0.1% SDS, 2 mM EDTA, 100 mM NaF, and 1 mM Na_3_VO_4_) containing protease inhibitors (1 mM PMSF, 0.2 trypsin inhibitory units/mL aprotinin, and 20 μg/mL leupeptin). Immunoblotting and immunoprecipitation were performed as previously described [67]. Chemiluminescent detection and quantification were performed using a luminescence image system (LAS-4000, Fujifilm).

### Purification of His-tagged vimentin

His-tagged vimentin proteins were expressed in BL21 (DE3) *Escherichia coli* by 0.5 mM isopropyl β-D-thiogalactopyranoside induction. The bacterial pellets were washed sequentially with cold PBS, 1% NP-40 lysis buffer, and RIPA lysis buffer. The bacteria were lysed in vimentin extraction buffer (7 M Urea, 34 mM PIPES, 1.4 mM MgCl_2_, 1.4 mM EDTA, and 5 mM β-mercaptoethanol) with pulsed sonication. The lysates were centrifuged at 15,000 × g for 10 min at 4 °C to remove debris. The supernatants were dialyzed three times with 200 mL of vimentin dialysis buffer (34 mM PIPES, 1.4 mM EDTA, and 5 mM β-mercaptoethanol) at 4 °C for 12 h and stored at −80 °C.

### In vitro polymerization of vimentin

Purified His-vimentin (0.3 mg/mL in 100 µL of dialysis buffer) was polymerized by the addition of 150 mM NaCl and incubation at 30 °C for 30 min, which was followed by centrifugation at 100,000 × g for 20 min. The pellets were redissolved in vimentin extraction buffer. An equal proportion of His-vimentin in the supernatant and pellet fractions was fractionated by SDS-PAGE and visualized with Coomassie blue stain. The amount of vimentin polymerization was measured using ImageJ software. To visualize the in vitro-polymerized His-vimentin with immunofluorescence staining, the His-vimentin proteins were polymerized and stained with anti-vimentin (V9, 1:200) at 4 °C for 90 min, then followed by Alexa Fluor 488-conjugated secondary antibody for another 90 min. An aliquot (50 µL) was dropped onto a glass slide, semidried at 37 °C, mounted in Anti-Fade Dapi-Fluoromount-G (SouthernBiotech), and visualized with a Zeiss ApoTome2 microscope imaging system.

### Cryo-electron microscopy

Purified His-vimentin proteins were centrifuged at 10,000 × g for 5 min at 4 °C, after which, the soluble His-vimentin proteins in the supernatants were polymerized at 30 °C for 30 min. A droplet of the polymerized vimentin (4 µL) was adsorbed onto a glow-discharged holey carbon grid for 1 min, and the excess fluid was removed with filter paper. A droplet (4 µL) of 16% uranyl acetate was then added and blotted. The grids with samples were subsequently plunge-frozen in ethane using a Cryoplunge 3 System (Gatan, Inc.). Images were recorded with a JEOL1400 transmission electron microscope using an accelerating voltage of 120 kV on a 4 K × 4 K CCD camera (Gatan 895).

### In vitro kinase assay

GFP-c-Src Y527F and its kinase-defective mutant were transiently expressed in HEK293 cells. The GFP-Src immunoprecipitates by anti-GFP (Roche) were washed three times with 1% NP-40 lysis buffer and twice with 20 mM Tris buffer, pH 7.4. Kinase reactions were performed in 40 μL of kinase buffer (50 mM Tris-HCl, pH 7.4, 50 mM MnCl_2_) containing 100 µM ATP and 0.5 μg purified His-vimentin protein at 25℃ for 30 min. The reaction was terminated with the SDS sample buffer, and the proteins were fractionated by SDS-PAGE and analyzed by immunoblotting using anti-PY.

### In vitro phosphatase assay

FLAG-SHP2 was transiently expressed in HEK293 cells and the cells were lysed in TBS buffer (1% Triton X-100, 50 mM Tris-HCl, pH 7.4, and 150 mM NaCl) containing protease inhibitors and subjected to centrifugation at 19,200 × g at 4℃ for 10 min. To precipitate FLAG-SHP2, the cell lysates were incubated with anti-FLAG M2 affinity resins (Sigma-Aldrich #A2220) for 2 h according to the manufacturer’s instructions. FLAG-SHP2 proteins were eluted from the resins with 100 μM 3x FLAG peptide (30 μL) (Sigma Aldrich #F4799). mCherry-vimentin was transiently co-expressed with GFP-Src Y527F in HEK293 cells. mCherry-vimentin was immunoprecipitated by anti-mCherry and used as the substrate for FLAG-SHP2. The purified FLAG-SHP2 proteins were incubated with immobilized mCherry-vimentin in 300 μL of phosphatase reaction buffer (25 mM Imidazole, pH 7.0, 2.5 mM EDTA, 50 mM NaCl, 5 mM DTT) at 4℃ for 2 h and gently mix by rotation. The mixture was then washed with RIPA buffer for three times, boiled for 3 min in SDS sample buffer, and the proteins were fractionated by SDS-PAGE. The tyrosine phosphorylation of mCherry-vimentin was analyzed by immunoblotting using anti-phosphotyrosine.

### Small GTPase activity assay

GTP-bound Rac in whole-cell lysates was pulled down by immobilized GST-PAK-CRIB in a GTPase assay buffer (10% glycerol, 50 mM Tris, pH 7.4, 100 mM NaCl, 1% NP-40, and 2 mM MgCl_2_) containing protease inhibitors. The washed complexes were analyzed by immunoblotting using an antibody specific to Rac1.

### Trans-well migration assay

MCF7 cells and NIH3T3 cells were collected by trypsinization and suspended in serum-free medium. The experiments were performed in Neuro Probe (Cabin John, MD, USA) 48-well chemotaxis chambers. For the MCF7 cells, the lower chamber was loaded with serum-free medium with type I collagen (10 µg/mL) and EGF (200 ng/mL). For the NIH3T3 cells, the lower chamber was loaded with serum-free medium with fibronectin (10 µg/mL) and PDGF (50 ng/mL). The cells (5 × 10^3^) in serum-free medium were added to the upper chamber. The lower and upper chambers were separated by a polycarbonate membrane (Poretics, Livermore, CA) with an 8-µm (MCF7) or 4-µm (NIH3T3) pore size. The MCF7 and NIH3T3 cells were allowed to migrate for 24 and 4 h, respectively, at 37 °C in a humidified atmosphere containing 5% CO_2_. The membranes were fixed in methanol for 10 min and stained with modified Giemsa stain for 1 h. The cells that migrated to the lower side of the membrane were counted under a light microscope. Each experiment was performed in triplicate.

### Wound healing assay

The wound-healing assay was performed with Culture-Insert wells (ibidi) coated with 10 µg/mL of collagen. MCF7 cells (3 × 10^4^) were grown as a monolayer for 18 h and then serum-starved for 24 h. A 500 ± 50 µm cell-free gap was generated, and the cells were treated with or without 200 ng/mL EGF. To record the healing process, the closure of the gap was visualized every 5 min for 6 h on a ZEISS observer D1 microscope (Axio Observer D1; Carl Zeiss).

### Immunofluorescence staining

Cells were fixed with 3% paraformaldehyde in 90% methanol at −20 °C for 30 min and permeabilized with 0.05% Triton X-100 for 10 min at room temperature. Slides were stained with primary antibodies for 90 min and followed by incubation with Alexa Fluor 488- or 546-conjugated secondary antibodies for 90 min. The primary antibodies, including anti-keratin18 (1:100), anti-vimentin (V9; 1:200), anti-Vav2 (1:50), anti-FLAG (1:500), and anti-mCherry (1:200), were used in this study. Coverslips were mounted in Anti-Fade Dapi-Fluoromount-G (SouthernBiotech). The images in Figs. 1a, 1c, 2b, 2c, and 5c were acquired on a LEICA epifluorescence microscope using a PL Fluotar 100 × /NA 1.3 oil objective. The images in Fig. 1b were acquired using a Carl Zeiss LSM510 microscope imaging system with a Zeiss Plan-Apochromat 63 × /NA 1.4 oil immersion objective. The images in Figs. 2a, 4c, 5a, 5b, 7d and Supp. Fig. 1b were acquired using a Zeiss ApoTome2 microscope imaging system with a Zeiss Plan-Apochromat 63 × or 100 × /NA 1.4 oil immersion objective. The images in Fig. 8c were acquired using a Carl Zeiss LSM880 microscope imaging system with a Zeiss α Plan-Apochromat 100 × /NA 1.46 oil DIC M27 Elyra objective. The images were cropped with Photoshop CS6 (Adobe) and assembled into figures with Illustrator CS6 (Adobe).

### Fluorescence recovery after photobleaching (FRAP)

MCF7 cells expressing mCherry-vimentin or its mutants were seeded on glass-bottomed dishes. FRAP measurements were performed under a confocal microscope equipped with two simultaneous laser scanners (FV1000, Olympus, Tokyo, Japan) in a 37 °C and 5% CO_2_ environment. A 50-mW 405-nm laser set at 100% was used through one scanner for 1 s to photobleach a 3-μm wide band across the cells, and the fluorescence images of mCherry-vimentin (543 nm laser excitation) were obtained over time for 60 min through the second scanner. For the FRAP analysis, the recovery of the relative fluorescence ratios in the cytosol was normalized to the same regions before photobleaching after background subtraction and analyzed using FLOWVIEW software (Olympus).

### Fluorescence loss in photobleaching (FLIP)

MCF7 cells expressing mCherry-vimentin were first seeded on glass-bottomed dishes. FLIP measurements were performed under a confocal microscope equipped with two simultaneous laser scanners (FV1000, Olympus, Tokyo, Japan) in a 37°C and 5% CO_2_ environment. One scanner was used to continuously bleach a 3-μm diameter region for 10 min using a 50-mW 405-nm laser set at 100% while fluorescence images of mCherry-vimentin (543-nm laser excitation) were taken at one minute intervals for 10 min through the second scanner. For the FLIP analysis, the loss of fluorescence opposite to the bleached regions was measured. The relative fluorescence ratio in the cytosol of the bleached cells was normalized to the same regions before photobleaching and after background subtraction using FLOWVIEW software (Olympus).

### Live cell imaging

NIH3T3 cells stably expressing mCherry-vimentin were treated with 50 ng/mL PDGF in the presence or absence 200 nM dasatinib. The cells were incubated in a micro-cultivation system with temperature and CO_2_ control devices (Carl Zeiss). The cells were monitored on an inverted microscope (Axio Observer; Carl Zeiss) using a EC Plan-NEOFLUAR 40 × NA 0.75 objective. Images were captured every 15 sec for 1–3 h using a digital camera (ORCA-Flash4.0 V2; Hamamatsu) and were processed by ZEISS ZEN2 image software.

### Mass spectrometry

MEF and MEF/v-Src cells were lysed in 1% NP-40 lysis buffer containing protease inhibitors. The lysates were centrifuged at 15,000 × g for 10 min at 4 °C. The pellets were solubilized with RIPA lysis buffer and centrifuged again at 15,000 × g for 10 min at 4 °C. The vimentin in the supernatant was immunoprecipitated with anti-vimentin, and the immunocomplexes were fractionated by SDS-PAGE and stained with Coomassie blue. Mass spectrometry for protein identification and phosphorylation sites was performed as described previously [71].

### Densitometric quantitation and statistics

A densitometric quantitation of the scanned images was performed using ImageJ 1.43 software (National Institutes of Health). Significance was determined by unpaired Student *t*-tests for two samples and two-way ANOVAs for grouped data. The significance levels are indicated by asterisks: *p < 0.05; **p < 0.01; and ***p < 0.001. Adobe Illustrator CS6 was used for preparing the figures.

## Supplementary information

Supplemental Information
