# Supplementary material for: Src and SHP2 coordinately regulate the dynamics and organization of vimentin filaments during cell migration
Source: Oncogene. 2019 Jan 29;38(21):4075–94. doi: 10.1038/s41388-019-0705-x (PMC6755999; doi:10.1038/s41388-019-0705-x)
Supplement: Supplementary file 1 — Supplemental Information [file 41388_2019_705_MOESM1_ESM.pdf]

Supplementary Information for

Src and SHP2 coordinately regulate the dynamics and organization of vimentin filaments during cell migration

Cheng-Yi Yang[1], Po-Wei Chang[1], Wen-Hsin Hsu[2], Hsuan-Chia Chang[3] Chien-Lin Chen[1], Chien-Chen Lai[4*], Wen-Tai Chiu[5*], and Hong-Chen Chen [2,3*]

[1]Department of Life Sciences, National Chung Hsing University, Taichung, Taiwan; [2]Cancer Progression Research Center, National Yang-Ming University, Taipei, Taiwan; [3]Institute of Biochemistry and Molecular Biology, National Yang-Ming University, Taipei, Taiwan; [4]Institute of Molecular Biology, National Chung Hsing University, Taichung, Taiwan; [5]Department of Biomedical Engineering, National Cheng Kung University, Tainan, Taiwan

This PDF file includes:

Supplementary Figure 1

Supplementary Figure 2

Supplementary Figure 3

Supplementary Figure 4

Supplementary Figure 5

Supplementary Figure 6

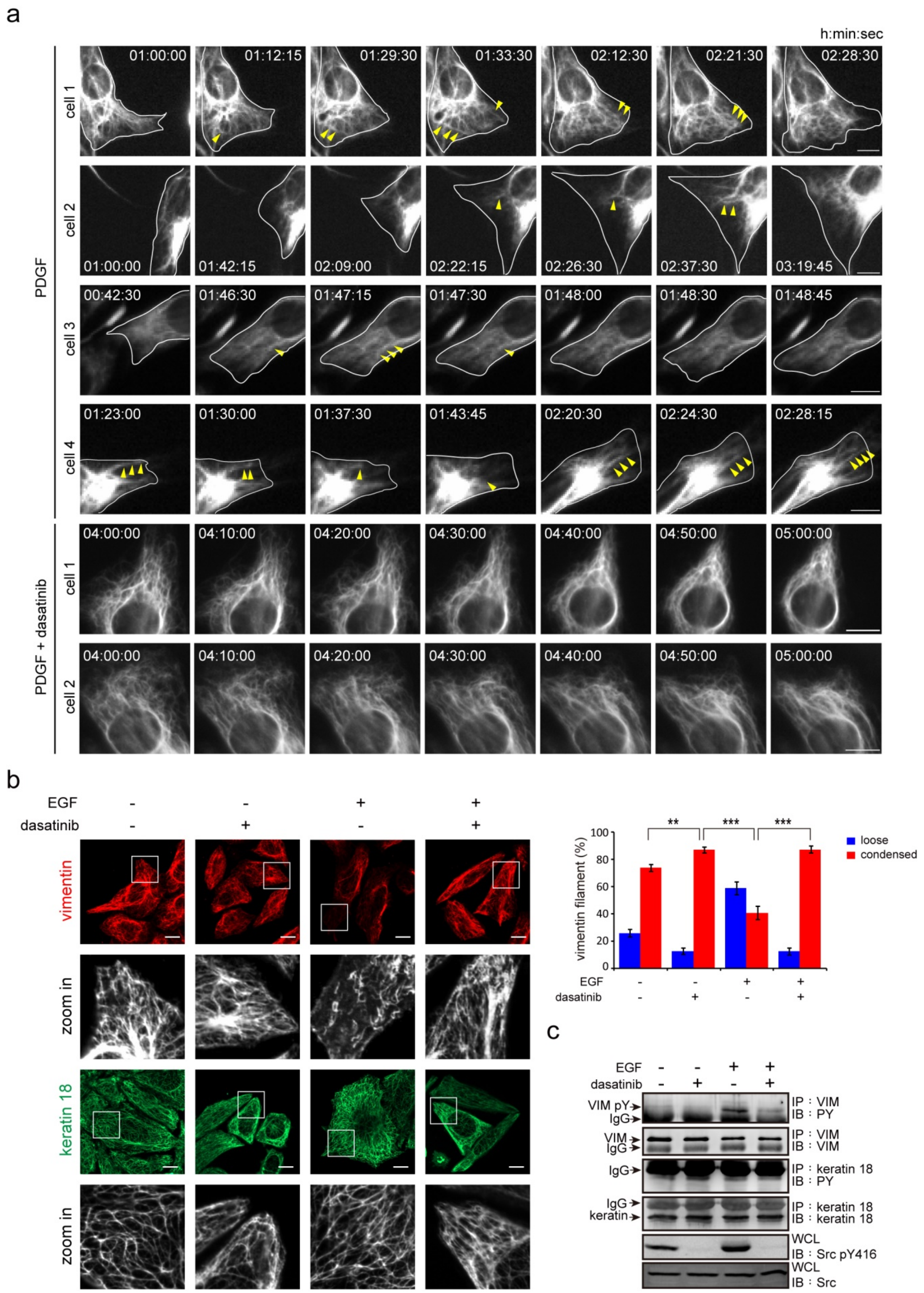

Figure S1. Src is important for the reorganization of VIFs in response to growth factor stimulation. a NIH3T3 cells stably expressing mCherry-vimentin were serum-starved and then treated with 50 ng/mL PDGF in the presence or absence of 200 nM dasatinib. The

reorganization of mCherry-vimentin were monitored with time-lapse microscopy. Images were captured every 15 sec. Representative images from four cells treated with PDGF and two cells treated with PDGF and dasatinib are shown. Arrowheads indicate the VIFs undergoing assembly or disassembly. Scale bars, 10 μm. **b** HeLa cells were serum-starved for 24 h and then treated with (+) or without (-) 200 ng/mL EGF in the presence or absence of 100 nM dasatinib for 6 h. The cells were fixed and stained for vimentin and keratin 18. The organization of the vimentin and keratin 18 was visualized under a Zeiss ApoTome2 microscope. Scale bars, 10 μm. The proportion of the total counted cells (n ≥ 300) with condensed or loose VIFs was determined. Values (means ± SD) are from three independent experiments. **P < 0.01, ***P < 0.001. **c** HeLa cells as described in (b) were lysed in RIPA buffer. Vimentin and keratin 18 were immunoprecipitated (IP) with anti-vimentin and anti-keratin 18, respectively, and the immunocomplexes were analyzed by immunoblotting (IB) with anti-PY. An equal amount of whole cell lysates was analyzed by immunoblotting with anti-Src and anti-Src pY416.

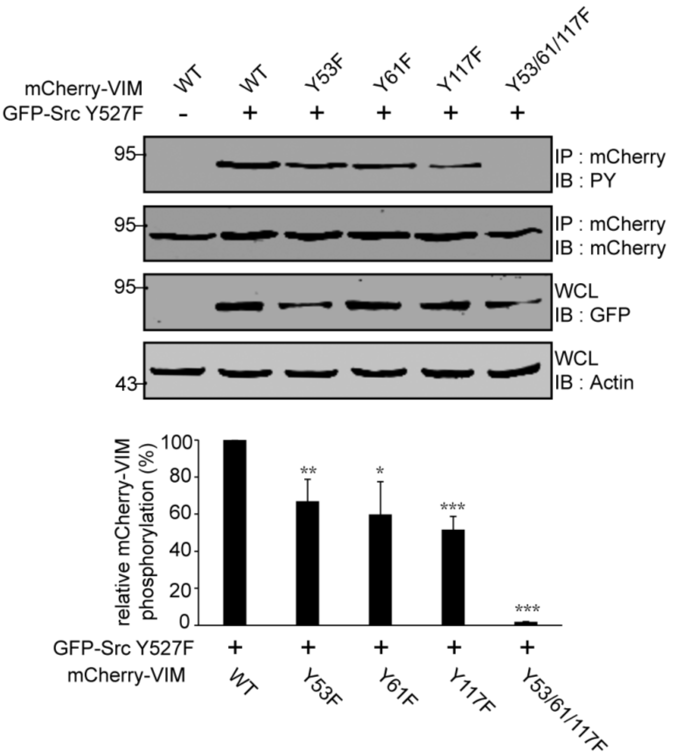

Figure S2. The Tyr53 and Tyr61 of vimentin are also phosphorylation sites for Src. mCherry-vimentin WT or mutants were transiently co-expressed with (+) or without (-) GFP-Src Y527F in HEK293 cells. mCherry-vimentin was immunoprecipitated (IP) with anti-mCherry and the immunocomplexes were analyzed by immunoblotting with anti-PY or anti-mCherry. The tyrosine phosphorylation of mCherry-VIM was quantified and expressed as a percentage relative to the WT level. Values (means ± SD) are from three independent experiments. *P < 0.05, **P < 0.01, ***P < 0.001.

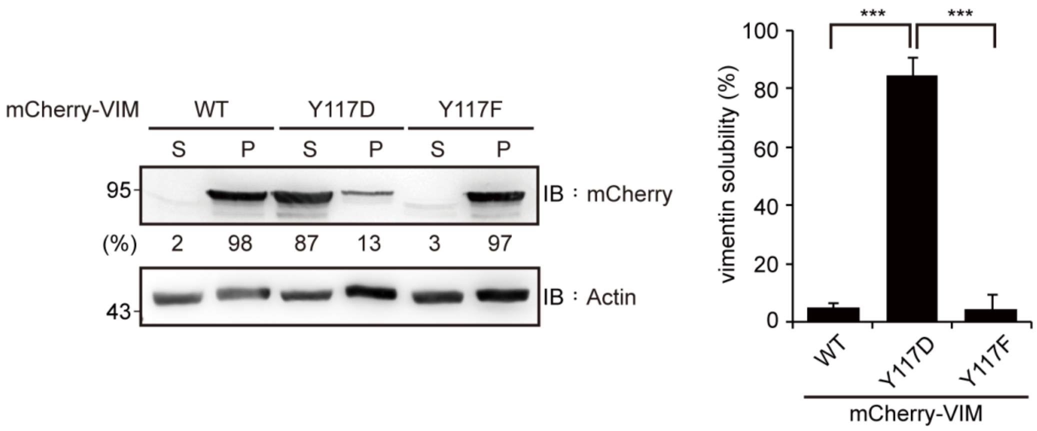

Figure S3. Phosphorylation of vimentin at Tyr117 increases its solubility in vivo. HeLa cells were transiently expressed with mCherry-vimentin and its mutants for 18 hours. The cells were lysed with 1% NP-40 lysis buffer and immediately centrifuged at 15,500 g in 4℃ for 10 mins. The supernatants (S) and precipitations (P) were separated. The precipitations were resolved with PIPA lysis buffer (0.1% SDS) by sonication and then centrifuged at 15,500 g in 4℃ for 10 mins. Equal amounts of whole-cell lysates were analyzed by immunoblotting with indicated antibodies. Western blots from the vimentin solubility percentage were measured by ImageJ. Values (means ± SD) are from three independent experiments. ***$P < 0.001$.

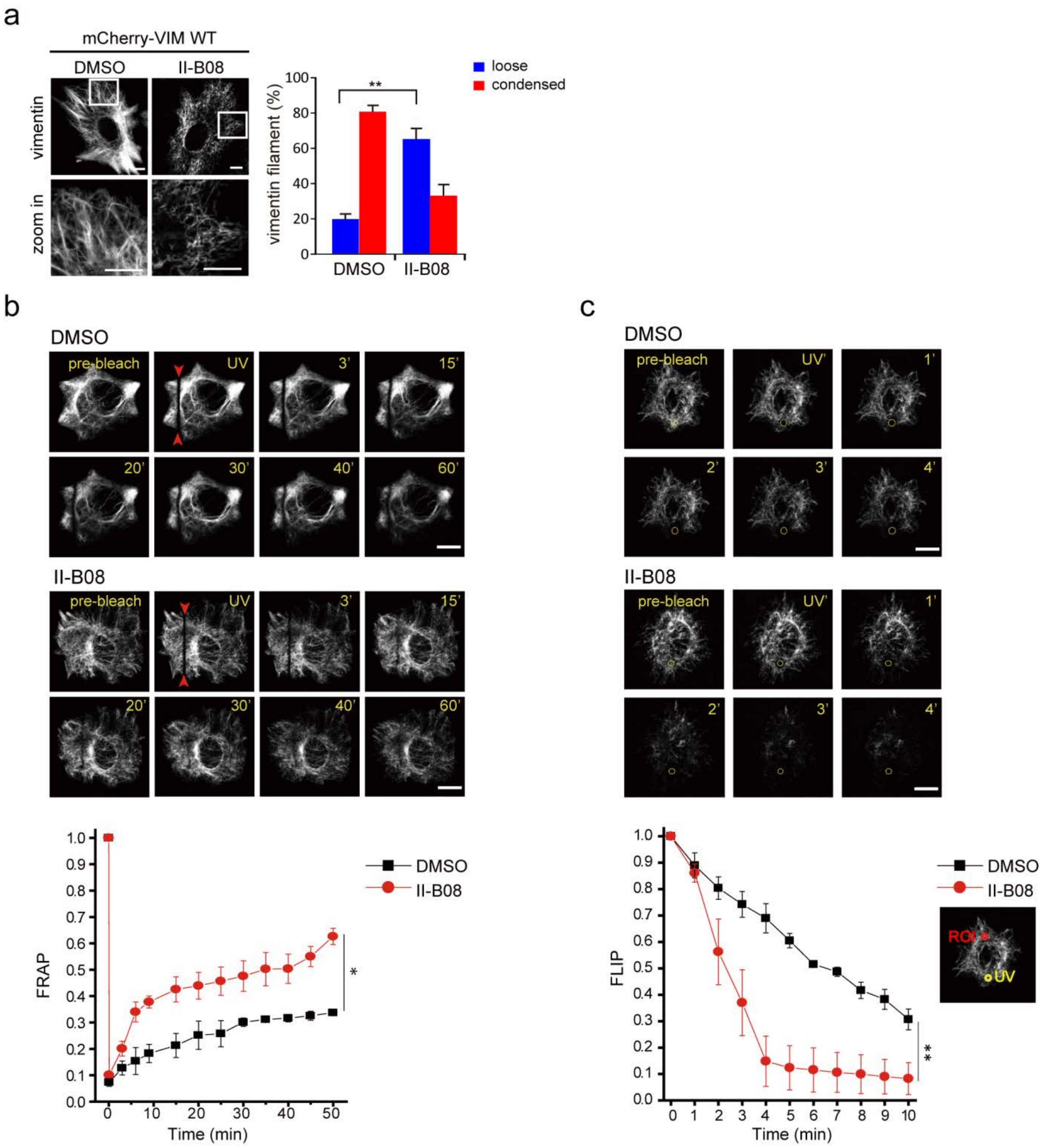

Figure S4. Inhibition of SHP2 by II-B08 promotes the dynamic of VIFs. a The MCF7 stably expressing mCherry-vimentin cells were grown on coverslips for 16 h and then treated with DMSO, SHP2 specific inhibitor II-B08 (10 μM) or dasatinib (100 nM) for 12 h. The cells were fixed and stained with anti-vimentin. The fluorescent images of vimentin were taken and shown by Apotome microscopy. The percentage of cells with condensed or loose VIFs in the total counted cells was determined (n ≥ 300). Values (means ± SD) are from three independent experiments. $*P < 0.05$, $**P < 0.01$. **b** Fluorescence recovery after photobleaching (FRAP) analysis was performed in MCF7 cells transiently expressing mCherry-vimentin-WT upon DMSO, II-B08 or dasatinib stimulation. The selected regions (indicated by red arrowheads) were photobleached once by laser at 405 nm for 1 second.

Representative images before bleaching (pre-bleach) and at different time points after bleaching from confocal fluorescence microscopy with excitation at 543 nm are shown. Bars, 10 μm. Fluorescence at the photobleached regions (indicated by red arrowheads) was measured and FRAP was calculated as the ratio of the initial fluorescence. Values (means ± SD) are from at least three independent experiments. Two-way ANOVA with Tukey's *post-hoc* test was used for comparison of DMSO *versus* II-B08 treatment and for comparison of DMSO *versus* dasatinib at each time point. *P < 0.05. **c** Fluorescence loss in photobleaching (FLIP) analysis was performed in MCF7 cells transiently expressing mCherry-vimentin-WT upon DMSO, II-B08 or dasatinib stimulation. The selected regions (yellow circle, 3-μm diameter) were photobleached by laser at 405 nm for 10 min. Representative images before bleaching (pre-bleach) and at different time points after bleaching from confocal fluorescence microscopy with excitation at 543 nm are shown. Bars, 10 μm. Fluorescence at the regions of interest (red circle with 3-μm diameter) was measured and FLIP was calculated as the ratio of the initial fluorescence. Values (means ± SD) are from at least 4 independent experiments. Two-way ANOVA with Tukey's *post-hoc* test was used for comparison of DMSO *versus* II-B08 treatment and for comparison of DMSO *versus* dasatinib at each time point. **P < 0.01.

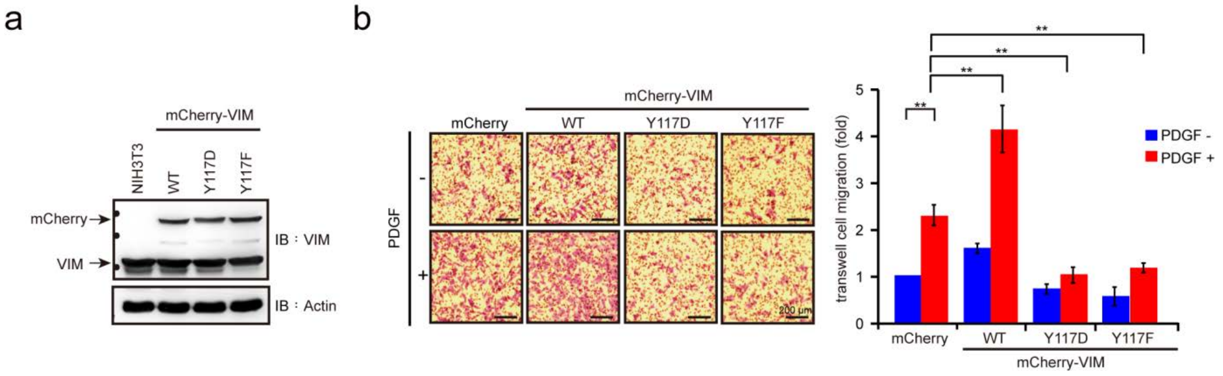

Figure S5. Proper dynamics of VIFs is important for NIH3T3 cell migration. a An equal amount of whole cells lysates from NIH3T3 cells stably expressing mCherry-VIM WT, Y117D and Y117F or mCherry alone as the control was analyzed by immunoblotting with anti-vimentin or anti-actin. **b** The NIH3T3 cells (5×10^3) were subjected to the trans-well cell migration assay in the presence (+) or absence (-) 50 ng/mL PDGF for 4 h. The cells migrated to the lower chamber were fixed, stained, and counted. Representative micrographs are shown. Scale bars, 200 μm. Data are expressed as fold relative to the level of the control cell in the absence of PDGF. Values (means ± SD) are from three independent experiments. **P < 0.01.

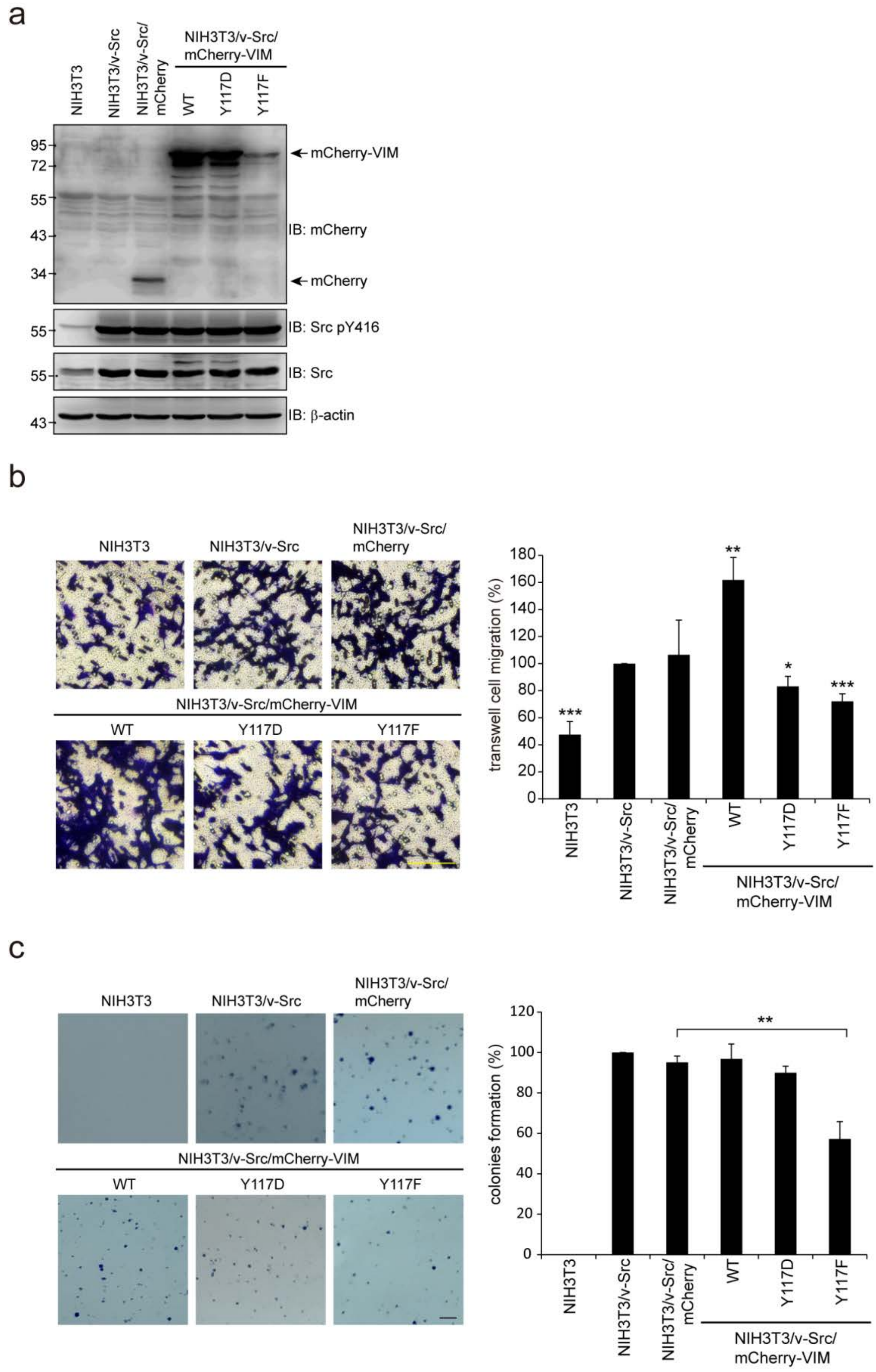

Figure S6. The Y117F mutant suppresses cell migration and anchorage-independent growth of vSrc-transformed NIH3T3 cells. a An equal amount of whole cells lysates from NIH3T3 cells, vSrc-transformed NIH3T3 (NIH3T3/vSrc) cells stably expressing mCherry-VIM WT, Y117D and Y117F or mCherry alone as the control was analyzed by

immunoblotting with indicated antibodies. **b** The cells were subjected to the trans-well cell migration assay for 6 h. Representative micrographs are shown. Scale bars, 100 μm. Values (means ± SD) are from three independent experiments. *P < 0.05, **P < 0.01, ***P < 0.001. **c** The cells were subjected to the soft agar colony formation assay. Cell colonies were fixed, stained, and counted after 7 days of incubation. Representative photographs of the experiments are shown. Scale bar, 1 mm. Values (means ± SD) are from three independent experiments. **P < 0.01.
